# Response to “Comment on ‘Exposure to Road Traffic Noise and Behavioral Problems in 7-Year-Old Children: A Cohort Study’”

**DOI:** 10.1289/ehp.1510851

**Published:** 2016-02-01

**Authors:** Dorrit Hjortebjerg, Anne Marie Nybo Andersen, Jeppe Schultz Christensen, Matthias Ketzel, Ole Raaschou-Nielsen, Jordi Sunyer, Jordi Julvez, Joan Forns, Mette Sørensen

**Affiliations:** 1 Danish Cancer Society Research Center, Copenhagen, Denmark; 2 Section of Social Medicine, Department of Public Health, University of Copenhagen, Denmark; 3 Department of Environmental Science, Aarhus University, Roskilde, Denmark; 4 Center for Research in Environmental Epidemiology, Barcelona, Spain; 5 Department of Genes and Environment, Norwegian Institute of Public Health, Oslo, Norway

We agree with Lezama et al. that the mental health of a parent may influence child behavior. Several family-level factors, including poor maternal mental health, have been demonstrated to pose a risk for behavioral problems in infancy and early childhood (Maggi et al. 2010). In our study, we had information on self-reported maternal mental health problems during pregnancy, and we adjusted for this covariate in the analysis. In response to the letter by Lezama et al. and to further assess this covariate we have conducted a sensitivity analysis in which we excluded women with mental health problems (*n* = 491). Exclusion of these women did not change the results of our study on either the total difficulties score (adjusted odds ration [OR] = 1.07; 95% confidence interval [CI]: 1.00, 1.14, compared with OR = 1.07; 95% CI: 1.00, 1.14 before exclusion) or the hyperactivity/inattention subscale (adjusted OR = 1.11; 95% CI: 1.03, 1.18, compared with adjusted OR = 1.10; 95% CI: 1.03, 1.18 before exclusion). This suggests that the associations between exposure to residential road traffic noise in early childhood and child behavior problems were not driven by maternal mental health problems.

We did not have information on the mental health of the father during pregnancy or of either parent after pregnancy, and our assessment of maternal mental health may not have fully captured mental health during pregnancy. Consequently, we cannot rule out that this may have affected the estimates in our study. There are, however, only very limited and inconclusive literature investigating the association between traffic noise and mental health among adults, which prevents us from commenting on the size of a possible effect modification by parental mental health.
